# Supplementary material for: Spectroscopic MRI-Based Biomarkers Predict Survival for Newly Diagnosed Glioblastoma in a Clinical Trial
Source: Cancers (Basel). 2023 Jul 7;15(13):3524. doi: 10.3390/cancers15133524 (PMC10340675; doi:10.3390/cancers15133524)
Supplement: Supplementary file 1 [file cancers-15-03524-s001.zip › cancers-2445957-supplementary.pdf]

Supplemental Table S1. Details about all 30 patients included in the original study.

Subject #	Age (years)	IDH status	MGMT status	Resection type	GTV3 volume (cc)	rENH volume (cc)
1	20.9	Mutant	MGMT+	GTR	48.2	1.6
2	57.9	WT	WT	STR	23.9	2.6
3	64.1	WT	MGMT+	GTR	65.0	0.2
4	59.0	WT	WT	GTR	19.3	1.4
5	55.9	WT	WT	GTR	18.5	0.0
6	71.6	WT	WT	GTR	16.5	0.0
7	38.1	WT	WT	GTR	17.1	0.3
8	55.1	WT	WT	GTR	6.4	0.5
9	60.1	WT	WT	Biopsy	20.0	15.4
10	64.3	WT	MGMT+	STR	20.2	2.2
11	65.4	WT	WT	GTR	9.5	0.5
12	55.7	WT	WT	GTR	0.9	0.6
13	70.9	WT	WT	Biopsy	2.4	1.4
14	54.2	WT	WT	Biopsy	63.4	12.1
15	24.8	Mutant	MGMT+	STR	34.8	2.9
16	59.6	WT	WT	GTR	5.4	0.9
17	66.8	WT	WT	STR	22.4	3.6
18	69.4	WT	WT	STR	24.6	3.9
19	57.4	WT	WT	STR	22.5	3.6
20	61.0	WT	WT	GTR	4.8	0.4
21	60.6	WT	MGMT+	STR	40.7	7.0
22	42.0	WT	WT	STR	4.1	0.4
23	59.7	WT	WT	Biopsy	33.6	11.3
24	58.8	WT	MGMT+	Biopsy	55.6	5.0
25	57.4	WT	MGMT+	GTR	7.6	4.9
26	68.2	WT	WT	STR	19.1	13.3
27	49.8	WT	MGMT+	STR	15.7	5.1
28	50.7	WT	WT	STR	19.4	2.0
29	42.8	WT	WT	GTR	31.6	0.5
30	69.6	WT	WT	STR	19.1	2.9

Abbreviations. IDH, isocitrate dehydrogenase; WT, wild type; MGMT, O^6-methylguanine-DNA methyltransferase; MGMT+, MGMT hypermethylated; GTR, gross total resection; STR, subtotal resection; GTV3, gross tumor volume 3; rENH, residual post-contrast enhancement.
